# Association between preoperative E/e′ and early left ventricular systolic recovery after coronary artery bypass grafting

**DOI:** 10.3389/fsurg.2026.1878735

**Published:** 2026-10-15

**Authors:** Jungsuk Choi, Hee Kyung Kim, Joon Kyu Kang

**Affiliations:** Department of Thoracic and Cardiovascular Surgery, Eunpyeong St. Mary's Hospital, College of Medicine, The Catholic University of Korea, Seoul, Republic of Korea

**Keywords:** coronary artery bypass graft, diastolic dysfunction, E/e′ ratio, left ventricular ejection fraction, transthoracic echocardiography

## Abstract

**Background:**

In patients with reduced or mildly reduced ejection fraction, the degree of systolic recovery after coronary artery bypass grafting (CABG) is heterogeneous. We assessed whether routine preoperative transthoracic echocardiography (TTE) indices—particularly E/e′—are associated with early postoperative improvement in left ventricular function.

**Methods:**

We retrospectively analyzed patients undergoing isolated CABG at a single tertiary center (April 2019 to July 2025). Eligibility required a preoperative left ventricular ejection fraction (LVEF) of <50%. Emergency/salvage operations, 30-day deaths, and patients without an evaluable early postoperative TTE were excluded. The primary outcome was an absolute increase in the LVEF of ≥10 percentage points. Because of the limited number of outcome events, the primary association analysis used a single-predictor logistic regression model with preoperative E/e′. Sensitivity analyses excluded patients with preoperative atrial fibrillation (AF) and modeled postoperative LVEF as a continuous outcome adjusted for baseline LVEF.

**Results:**

Of 75 otherwise clinically eligible patients, three lacked an evaluable early postoperative TTE, leaving 72 patients for outcome analysis; 17 (23.6%) achieved an LVEF increase of ≥10 percentage points. A higher preoperative E/e′ was associated with early LVEF improvement (OR 1.155 per 1-unit increase; 95% CI 1.04–1.28; *p* = 0.005; *N* = 66; 16 events). The apparent AUC was 0.700; after 1,000-resample bootstrap internal validation, the estimated optimism was 0.004 and the optimism-corrected AUC was 0.696 (95% bootstrap CI 0.526–0.885). The association persisted after excluding patients with AF (OR 1.14; 95% CI 1.02–1.27; *p* = 0.025). In a sensitivity analysis using postoperative LVEF as a continuous outcome, E/e′ remained associated with postoperative LVEF after adjustment for baseline LVEF (*β *= 0.31 percentage points per 1-unit increase; 95% CI 0.01–0.61; *p* = 0.042).

**Conclusion:**

A higher preoperative E/e′ was associated with early improvement in LV systolic function after CABG among patients with a preoperative LVEF of <50% who survived to undergo early postoperative echocardiographic assessment. Given the small number of events and the single-center retrospective design, these findings are hypothesis-generating and require external validation.

## Introduction

Left ventricular ejection fraction (LVEF) is the most widely used quantitative index of left ventricular systolic function and a central determinant of prognosis in ischemic heart disease. Contemporary international guidance classifies systolic impairment by LVEF ranges—reduced EF (typically <40%) and mildly reduced EF (41%–49%)—with preserved EF defined as ≥50%. These thresholds anchor risk stratification and therapeutic decision-making in daily practice (1, 2). In patients undergoing surgical revascularization, both the baseline level of EF and the magnitude of postoperative change carry prognostic significance. Observational data indicate that an absolute increase in the LVEF of ≥10 percentage points after CABG is associated with improved long-term outcomes (3–6). Meaningful improvement is uncommon when preoperative EF is preserved, consistent with contemporary classifications of systolic function (1, 2). Accordingly, we restricted the analytic cohort to patients with a preoperative EF of <50% to minimize ceiling effects and enhance the clinical interpretability of a ≥ 10-point improvement (1, 2). Beyond prognostication, randomized evidence demonstrates that CABG improves survival rates compared with medical therapy in ischemic left ventricular dysfunction (7). Yet, the degree of EF recovery is heterogeneous: Some patients exhibit substantial systolic improvement, while others fail to recover despite anatomically complete revascularization. Moreover, analyses from the STICH program suggest that conventional myocardial viability testing does not reliably identify subgroups with differential survival benefits from CABG, nor does it consistently predict functional recovery after adjustment (7, 8). These observations underscore the need for simple, widely available preoperative markers that can be obtained from routine testing. Preoperative echocardiography has also been associated with perioperative risk stratification in CABG patients (19). Among echocardiographic variables, E/e′—a guideline-endorsed surrogate of left ventricular filling pressure—offers biological plausibility as a predictor of postoperative functional change: Elevated preoperative filling pressures may reflect load-dependent, potentially reversible dysfunction in hibernating or stunned myocardium (20), while very low E/e′ may accompany fixed scar with limited reserve (2, 9). Furthermore, related parameters (e.g., LV s′ and LAVI) capture longitudinal systolic function and chronic diastolic load, which may modulate the likelihood and extent of post-revascularization improvement (2, 10, 11). In this context, echocardiographic markers obtained from routine transthoracic echocardiography (TTE) provide an attractive, pragmatic alternative to specialized viability imaging for everyday CABG candidates.

### Objective

We sought to identify preoperative clinical and echocardiographic characteristics associated with early postoperative improvement in systolic function after CABG, with a particular focus on the association between preoperative E/e′ and an absolute LVEF increase of ≥10 percentage points.

## Materials and methods

### Study design and patient selection

This retrospective cohort study was conducted at a single tertiary center and approved by the Institutional Review Board (IRB No. PC25RISI0191). We screened 342 patients who underwent CABG between April 2019 and July 2025. After excluding concomitant procedures (*n* = 28), 314 isolated CABG cases remained and 129 had a preoperative LVEF of <50%. Emergency/salvage operations (*n* = 47) and 30-day deaths (*n* = 7) were excluded, leaving 75 clinically eligible patients. Emergency and salvage CABG represent clinically distinct, higher-risk settings (17, 18). Three additional patients lacked an evaluable early postoperative TTE and could not be classified for the primary outcome, yielding a final outcome analysis cohort of 72 patients. The patient selection process is summarized in Figure 1. The requirement for written informed consent was waived because of the retrospective design and use of de-identified data.

**Figure 1:**
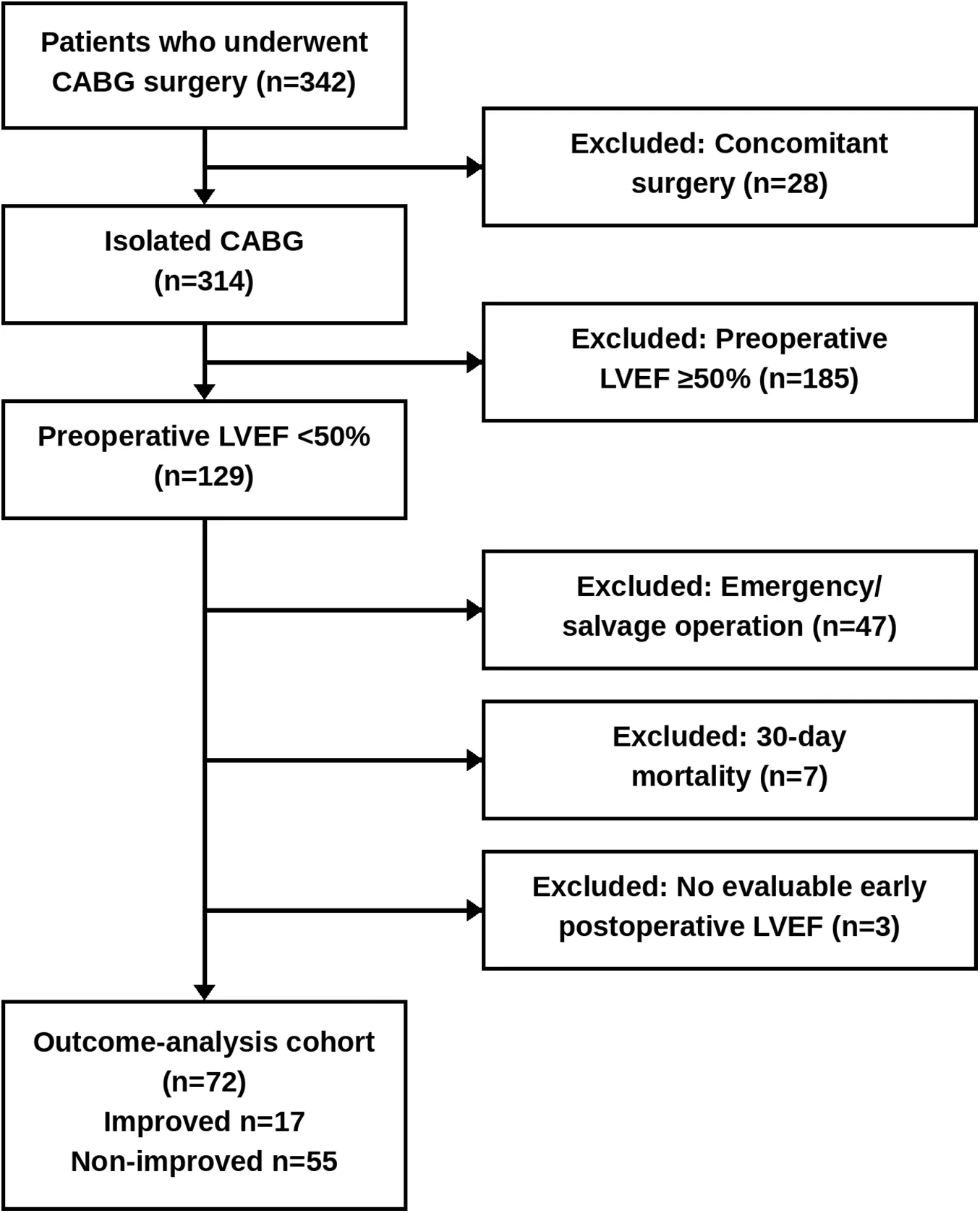
A study flow diagram. Of 342 patients who underwent CABG, 28 who underwent concomitant surgery were excluded, leaving 314 patients who underwent isolated CABG. Among 129 patients with preoperative LVEF <50%, 47 who underwent emergency/salvage operations and 7 who died within 30 days were sequentially excluded, leaving 75 clinically eligible patients. Three patients without an evaluable early postoperative LVEF were further excluded, resulting in a final outcome analysis cohort of 72 patients (Improved, *n* = 17; Non-improved, *n* = 55).

### Data collection and definitions

Clinical and echocardiographic variables were retrieved from the electronic medical record and routine echocardiography reports. Preoperative TTE parameters included LV end-diastolic diameter, LV end-systolic diameter, LVEF, E/e′, LV s′, LV mass index, relative wall thickness, left atrial volume index, deceleration time, and functional mitral regurgitation grade. Early postoperative TTE was performed at a median of 6 postoperative days [IQR 4–7; range 2–30]. Because this was a retrospective analysis of routinely acquired examinations, granular acquisition metadata needed to standardize heart rate/loading conditions, beat averaging in AF, reader blinding, and inter-/intraobserver reproducibility were not consistently available; this is acknowledged as a limitation.

### Outcome definition

The primary outcome was early postoperative improvement in LV systolic function, defined as an absolute increase in the LVEF (*Δ*LVEF) of  ≥ 10 percentage points from baseline on early postoperative TTE. Patients without an evaluable postoperative LVEF were not assigned to either outcome category. Early postoperative TTE was performed at a median of approximately 6 postoperative days (range, 2–30 days).

### Statistical analysis

Continuous variables were summarized as mean ± SD or median [IQR], as appropriate. Between-group comparisons used Student's t-test or the Mann–Whitney U test for continuous variables and the *χ*^2^ test or Fisher's exact test for categorical variables, as appropriate. Missing data were handled by an available-case analysis for descriptive and univariable comparisons; no imputation was performed, and the number of available observations is reported where relevant.

Because only 17 patients met the primary outcome and one of these patients lacked an E/e′ measurement, model complexity was intentionally restricted. The primary association analysis was a single-predictor logistic regression evaluating preoperative E/e′. Discrimination was summarized by the apparent ROC-AUC. Internal validation used 1,000 non-parametric bootstrap resamples; optimism was estimated as the mean difference between model performance in each bootstrap sample and the performance of that bootstrap-fitted model in the original sample. Discrimination and internal validation results were reported with reference to relevant TRIPOD reporting recommendations (21). The optimism-corrected AUC was calculated by subtracting the mean optimism from the apparent AUC, and a percentile bootstrap 95% CI was obtained from the corresponding optimism-corrected bootstrap distribution. Calibration was summarized by the bootstrap-corrected calibration intercept and slope; the Brier score was also calculated as an overall measure of probabilistic accuracy. The previously specified five-variable model (E/e′, LV s′, deceleration time, age, and preoperative AF) was retained only as an exploratory adjusted analysis because 16 complete-case events were insufficient for stable conventional multivariable modeling. Sensitivity analyses excluded patients with AF and, separately, modeled postoperative LVEF as a continuous outcome adjusted for baseline LVEF to address mathematical coupling and room-for-improvement bias. A subgroup analysis restricted to a baseline LVEF of <40% is provided in Supplementary Table S1.

## Results

### Patient characteristics

Baseline patient characteristics are summarized in Table 1. Of 314 isolated CABG patients, 75 met the clinical eligibility criteria after excluding emergency/salvage operations and 30-day deaths. Three patients lacked an evaluable early postoperative LVEF and could not be classified for the primary outcome, leaving 72 patients for outcome analysis. Seventeen (23.6%) were assigned to the Improved group and 55 (76.4%) to the Non-improved group. Early postoperative TTE was performed at a median of 6 days [IQR 4–7; range 2–30] after CABG.

**Table 1:** Baseline patient characteristics (overall; *N* = 72).

Variables		Value
Age, years		66.89 (±12.92, 6.00–86.00); *n* = 72
Male, *n* (%)		56/72 (77.8)
Diabetes, *n* (%)		41/72 (56.9)
Hypertension, *n* (%)		47/72 (65.3)
Hyperlipidemia, *n* (%)		23/72 (31.9)
Smoking (current/ever), *n* (%)		9/72 (12.5)
CVA/TIA, *n* (%)		9/72 (12.5)
Atrial fibrillation, *n* (%)		10/72 (13.9)
Prior PCI, *n* (%)		20/72 (27.8)
Creatinine, mg/dL		1.91 (±2.76, 0.49–17.55); *n* = 72
GFR, mL/min/1.73m^2^		67.36 (±32.93, 3.00–118.00); *n* = 72
NYHA class	I, *n* (%)	12/72 (16.7)
	II, *n* (%)	38/72 (52.8)
	III, *n* (%)	18/72 (25.0)
	IV, *n* (%)	4/72 (5.6)

Continuous variables are presented as mean ± SD (min–max); categorical variables are presented as *n*/*N* (%). Available-case analysis was applied; denominators may vary. Echocardiographic and preoperative group comparisons are presented in Table 2.

PCI, percutaneous coronary intervention; CVA, cerebrovascular accident; TIA, transient ischemic attack; GFR, glomerular filtration rate.

**Table 2:** Preoperative echocardiographic and clinical variables: improved (*n* = 17) vs. non-improved (*n* = 55).

Variable	Improved (*Δ*EF ≥10%; *n* = 17)	Not improved (*Δ*EF <10%; *n* = 55)	*P*-value
Baseline LVEF, %	27.9 [19.7, 33.0] (*n* = 17)	38.7 [28.9, 43.8] (*n* = 55)	0.005
LVEDD, mm	47.8 [44.2, 54.0] (*n* = 17)	52.5 [49.0, 55.3] (*n* = 54)	0.103
LVESD, mm	41.0 [36.1, 47.0] (*n* = 17)	43.4 [38.1, 46.2] (*n* = 54)	0.782
E/e′	15.7 [9.8, 22.1] (*n* = 16)	10.5 [8.4, 13.0] (*n* = 50)	0.017
LV s′, cm/s	4.9 [3.8, 5.3] (*n* = 16)	6.0 [5.2, 7.3] (*n* = 50)	0.009
Deceleration time, ms	174.5 [143.8, 190.9] (*n* = 16)	191.9 [146.6, 224.8] (*n* = 50)	0.234
LV mass index, g/m^2^	96.8 [86.7, 124.6] (*n* = 17)	114.3 [96.4, 137.4] (*n* = 54)	0.309
Relative wall thickness	0.42 [0.34, 0.48] (*n* = 17)	0.38 [0.33, 0.42] (*n* = 54)	0.214
LAVI, mL/m^2^	46.9 [35.5, 49.6] (*n* = 17)	34.7 [28.8, 44.6] (*n* = 54)	0.036
Male, *n* (%)	11/17 (64.7)	45/55 (81.8)	0.183
Age, years	69.0 [66.0, 78.0] (*n* = 17)	67.0 [58.0, 75.0] (*n* = 55)	0.196
NYHA class (ordinal)	3.0 [2.0, 3.0] (*n* = 17)	2.0 [2.0, 2.0] (*n* = 55)	0.044
Diabetes, *n* (%)	9/17 (52.9)	32/55 (58.2)	0.783
Hypertension, *n* (%)	10/17 (58.8)	37/55 (67.3)	0.568
Hyperlipidemia, *n* (%)	7/17 (41.2)	16/55 (29.1)	0.383
Smoking (current/ever), *n* (%)	2/17 (11.8)	7/55 (12.7)	1.000
CVA/TIA, *n* (%)	2/17 (11.8)	7/55 (12.7)	1.000
Creatinine, mg/dL	1.0 [0.8, 1.2] (*n* = 17)	1.0 [0.8, 1.3] (*n* = 55)	0.884
GFR, mL/min/1.73 m^2^	66.0 [54.0, 79.0] (*n* = 17)	75.0 [51.0, 94.0] (*n* = 55)	0.458
Atrial fibrillation, *n* (%)	5/17 (29.4)	5/55 (9.1)	0.049
Prior PCI, *n* (%)	3/17 (17.6)	17/55 (30.9)	0.365
No. of diseased vessels	3.0 [3.0, 3.0] (*n* = 17)	3.0 [2.0, 3.0] (*n* = 55)	0.891

Continuous variables are presented as median [IQR]; categorical variables are presented as *n*/*N* (%). Group comparisons used Mann–Whitney *U* (continuous) and *χ*^2^ or Fisher's exact tests (categorical), as appropriate. Available-case analysis was performed; denominators may vary.

LVEDD, left ventricular end-diastolic diameter; LVESD, left ventricular end-systolic diameter; LAVI, left atrial volume index; LV s′, tissue Doppler systolic velocity; E/e′, ratio of mitral inflow E wave to tissue Doppler e′; CVA, cerebrovascular accident; TIA, transient ischemic attack; GFR, glomerular filtration rate; PCI, percutaneous coronary intervention; IQR, interquartile range.

### Preoperative echocardiographic differences

Patients in the Improved group had a lower baseline LVEF than those in the Non-improved group (27.9% [19.7–33.0] vs. 38.7% [28.9–43.8], *p* = 0.005). E/e′ was higher in the Improved group (15.7 [9.8–22.1], *n* = 16 vs. 10.5 [8.4–13.0], *n* = 50; *p* = 0.017), whereas LV s′ was lower (4.9 [3.8–5.3], *n* = 16 vs. 6.0 [5.2–7.3], *n* = 50; *p* = 0.009). LAVI was also higher (*p* = 0.036). Preoperative AF was present in 5 of 17 (29.4%) versus 5 of 55 (9.1%; Fisher's exact *p* = 0.049).

### Intraoperative and postoperative findings

Intraoperative and early postoperative variables are summarized in Table 3. These comparisons were exploratory and were not interpreted as evidence that perioperative factors were unrelated to early LV recovery.

**Table 3:** Intraoperative and early postoperative variables: improved vs. not improved.

Variable	Improved (*Δ*EF ≥10%; *n* = 17)	Not improved (*Δ*EF <10%; *n* = 55)	*P*-value
Intraoperative variables			
Anesthesia time, min	180.0 [170.0, 230.0]	190.0 [150.0, 215.0]	0.853
Operation time, min	140.0 [120.0, 185.0]	140.0 [105.0, 172.5]	0.760
CPB time, min	0.0 [0.0, 0.0]	0.0 [0.0, 0.0]	0.823
No. of anastomoses	2.0 [1.0, 3.0]	2.0 [1.0, 3.0]	0.945
LAD diameter, mm	1.5 [1.5, 1.5] (*n* = 17)	1.5 [1.5, 1.8] (*n* = 54)	0.208
Transfusion—RBC, units	6.0 [4.0, 7.0]	5.0 [4.0, 7.8] (*n* = 54)	0.769
Transfusion—FFP, units	3.0 [3.0, 4.0]	3.0 [3.0, 5.0]	0.212
Transfusion—Platelet, units	6.0 [6.0, 12.0]	6.0 [6.0, 6.0] (*n* = 54)	0.829
Transfusion—Cryo, units	0.0 [0.0, 0.0]	0.0 [0.0, 4.0]	0.292
On-pump CPB, *n* (%)	3/17 (17.6)	10/55 (18.2)	1.000
LIMA-to-LAD, *n* (%)	17/17 (100.0)	55/55 (100.0)	N/A
Hybrid CABG, *n* (%)	1/17 (5.9)	9/54 (16.7)	0.433
Early postoperative variables			
Intubation time, h	6.0 [4.4, 12.1] (*n* = 16)	7.0 [4.0, 14.0] (*n* = 55)	0.825
Troponin-I (6 h), ng/mL	1.6 [0.4, 3.1]	1.0 [0.3, 3.3]	0.710
Troponin-I (18 h), ng/mL	1.1 [0.4, 5.8]	1.0 [0.3, 4.2] (*n* = 54)	0.711
Troponin-I (30 h), ng/mL	1.0 [0.3, 5.7]	0.9 [0.2, 3.8] (*n* = 54)	0.522
Troponin-I (48 h), ng/mL	0.6 [0.2, 2.3]	0.7 [0.1, 1.8] (*n* = 54)	0.788
Ultrafast-track extubation, *n* (%)	2/17 (11.8)	5/54 (9.3)	0.670
Postoperative norepinephrine, *n* (%)	1/16 (6.2)	2/50 (4.0)	1.000
Postoperative amiodarone, *n* (%)	6/16 (37.5)	9/49 (18.4)	0.170

Continuous variables are presented as median [IQR]; categorical variables are presented as *n*/*N* (%). Group comparisons used Mann–Whitney *U* (continuous) and *χ*^2^ or Fisher's exact tests (categorical), as appropriate. Available-case analysis was applied; denominators may vary by variable.

CPB, cardiopulmonary bypass; LIMA, left internal mammary artery; LAD, left anterior descending artery; FFP, fresh frozen plasma; Cryo, cryoprecipitate; IQR, interquartile range.

### Univariable associations

In the primary single-predictor logistic regression analysis (*N* = 66; 16 events), a higher preoperative E/e′ was associated with greater odds of early LVEF improvement (OR 1.155 per 1-unit increase; 95% CI 1.044–1.278; *p* = 0.005). The apparent AUC was 0.700. The ROC curve for the primary E/e′ model is shown in Figure 2. With 1,000-resample bootstrap internal validation, the mean estimated optimism was 0.0043, yielding an optimism-corrected AUC of 0.696 (95% bootstrap CI 0.526–0.885). The bootstrap-corrected calibration intercept was 0.01 (95% bootstrap CI −0.57 to 0.75), and the calibration slope was 1.17 (95% bootstrap CI 0.47–3.30); the apparent Brier score was 0.154. The wide intervals, particularly for calibration, reflected substantial uncertainty related to the small number of events, and therefore, model performance was interpreted cautiously.

**Figure 2:**
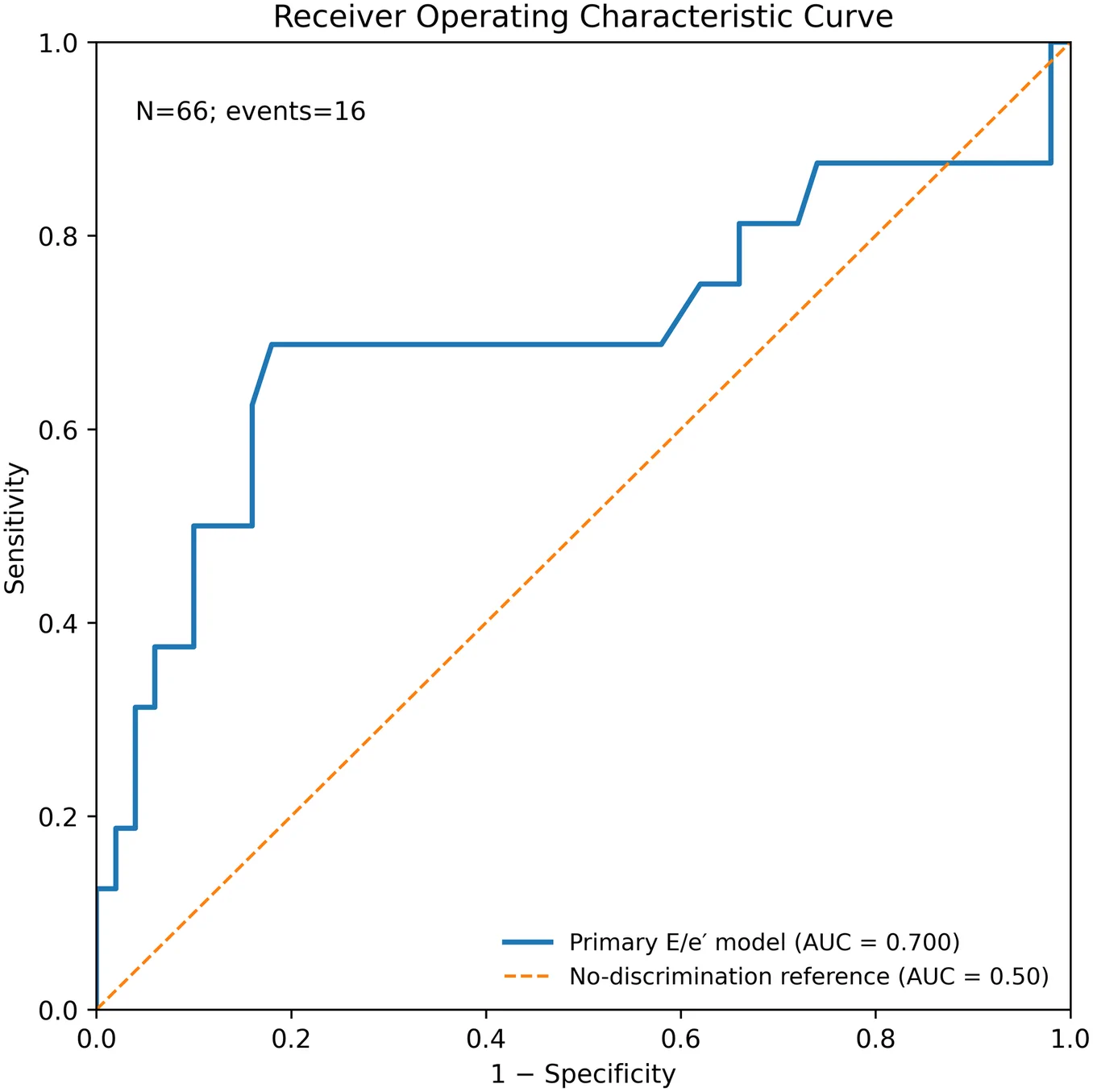
Receiver operating characteristic (ROC) curve for the primary E/e′ association model. Single-predictor logistic regression with preoperative E/e′; *N* = 66; events = 16. Apparent AUC = 0.700; mean bootstrap optimism = 0.0043; optimism-corrected AUC = 0.696 (95% bootstrap CI 0.526–0.885). Bootstrap-corrected calibration intercept = 0.01 and calibration slope = 1.17. Internal validation used 1,000 bootstrap resamples.

#### Exploratory adjusted and sensitivity analyses

In an exploratory complete-case model containing E/e′, LV s′, deceleration time, age, and preoperative AF (*N* = 66; 16 events), E/e′ remained associated with the outcome (adjusted OR 1.136; 95% CI 1.021–1.264; *p* = 0.019; apparent AUC = 0.765). The exploratory multivariable logistic regression results are presented in Table 4. This model was considered exploratory because of the low events-per-variable ratio. After excluding patients with preoperative AF, the E/e′ association persisted (*N* = 57; 12 events; OR 1.135; 95% CI 1.016–1.268; *p* = 0.025). In the baseline-LVEF-adjusted linear regression sensitivity analysis (*N* = 66), E/e′ remained associated with postoperative LVEF (*β* = 0.31 percentage points per 1-unit increase; 95% CI 0.01–0.61; *p* = 0.042), while baseline LVEF was also strongly associated with postoperative LVEF (*β* = 0.71; 95% CI 0.53–0.90; *p* < 0.001).

**Table 4:** Exploratory multivariable logistic regression analysis for early LVEF improvement.

Predictor	Adjusted OR	95% CI	*P*-value
E/e′ (per 1 unit)	1.136	1.021–1.264	0.019
LV s′ (per 1 cm/s)	0.822	0.558–1.211	0.322
Deceleration time, DT (per 1 ms)	0.995	0.984–1.005	0.330
Age (per 1 year)	1.015	0.961–1.072	0.587
Preoperative AF (yes vs. no)	2.148	0.371–12.424	0.393

Primary analysis: single-predictor E/e′ logistic regression (*N* = 66; events = 16). The five-variable complete-case model is exploratory because of the limited event count. ORs are reported with 95% CIs.

## Discussion

In this retrospective cohort of patients with a preoperative LVEF of <50% undergoing isolated CABG, a higher preoperative E/e′ was associated with a greater likelihood of early LVEF improvement. The association persisted in analyses excluding AF and when postoperative LVEF was analyzed as a continuous outcome with adjustment for baseline LVEF. These findings support E/e′ as a potentially informative preoperative marker, but they do not establish causality or a clinically actionable prediction threshold.

The Improved group had a substantially lower baseline LVEF, emphasizing the room-for-improvement effect inherent in a change-score outcome. Therefore, we did not include baseline LVEF in the primary change-score logistic model and instead performed a complementary analysis of postoperative LVEF adjusted for baseline LVEF. The persistence of the E/e′ association in that analysis reduces, but does not eliminate, concerns that the primary finding reflects mathematical coupling or baseline severity alone.

The present results should not be used to define a clinical E/e′ cutoff or to counsel individual patients regarding the probability of functional recovery. The modest AUC and wide uncertainty from internal validation indicate that E/e′ alone is insufficient as a stand-alone prediction tool. Therefore, the observed association should be considered hypothesis-generating and evaluated in larger, externally validated cohorts.

E/e′ is influenced by LV relaxation, filling pressure, rhythm, heart rate, and loading conditions. Thus, a higher E/e′ may identify a distinct hemodynamic phenotype among patients with ischemic LV dysfunction, but the mechanism linking E/e′ to early systolic recovery cannot be established from this retrospective study. The persistence of the association after excluding AF provides some reassurance regarding rhythm-related confounding, although residual confounding remains possible.

Global longitudinal strain (GLS) is a more sensitive marker of LV systolic function than LVEF and benefits from standardized acquisition and analysis guidance from professional consensus, improving reproducibility across vendors and laboratories (12). Beyond general prognostic utility, a meta-analysis has shown that GLS outperforms LVEF in risk prediction across cardiovascular populations (13). In surgical revascularization cohorts, preoperative or perioperative GLS independently predicts outcomes after CABG, with some studies suggesting that post-CPB GLS may provide the strongest postoperative prognostic signal (14–16). In our cohort, GLS could not be evaluated because speckle-tracking echocardiography was not consistently performed, leaving too few analyzable studies for robust modeling. Future work should prospectively incorporate standardized GLS protocols and prespecified quality control to test whether adding GLS improves discrimination and calibration over E/e′ alone (12–16).

## Conclusion

Among patients with a preoperative LVEF of <50% who survived and underwent early postoperative TTE after isolated CABG, a higher preoperative E/e′ was associated with early improvement in LV systolic function. This association persisted in sensitivity analyses addressing AF and baseline LVEF. Given the small number of events, modest discrimination, retrospective single-center design, and survivorship inherent in the analyzed cohort, these findings should be regarded as hypothesis-generating and require external validation.

## Limitations

This study has several limitations. First, its retrospective single-center design limits causal inference and generalizability. Second, the small sample size and limited number of outcome events constrained model complexity; the five-variable adjusted model is therefore exploratory and potentially unstable. Third, three otherwise eligible patients lacked early postoperative TTE and could not be classified for the primary outcome, while additional echocardiographic variables had sporadic missingness; available-case and complete-case analyses may be biased if the missingness was informative. Fourth, 30-day deaths were excluded because early echocardiographic recovery could not be meaningfully evaluated, introducing survivorship bias; the findings apply to patients who survived to early postoperative TTE rather than to all CABG patients with reduced LVEF. Fifth, baseline LVEF was lower in the Improved group, raising concerns about room-for-improvement and mathematical coupling, although the baseline-adjusted postoperative LVEF sensitivity analysis yielded a consistent E/e′ association. Sixth, E/e′ is sensitive to rhythm, heart rate, loading conditions, and Doppler acquisition; granular retrospective acquisition metadata, reader blinding, and reproducibility measures were not consistently available in this study. Seventh, preoperative BNP/NT-proBNP and other biomarkers were not consistently available, although serial postoperative troponin-I measurements were examined. Finally, early TTE occurred at a median of 6 postoperative days [IQR 4–7; range 2–30]; therefore, this study addresses early postoperative functional change rather than longer-term LV remodeling.

## Data Availability

The original contributions presented in the study are included in the article/Supplementary Material, further inquiries can be directed to the corresponding author.
