# Supplementary material for: Association between preoperative E/e′ and early left ventricular systolic recovery after coronary artery bypass grafting
Source: Front Surg. 2026 Oct 15;13:1878735. doi: 10.3389/fsurg.2026.1878735 (PMC13716754; doi:10.3389/fsurg.2026.1878735)
Supplement: Supplementary file 1 [file Table1.docx]

**Supplementary Table S1. Univariable comparisons in the subgroup with preoperative LVEF <40%**

*(corrected outcome-analysis cohort; N=46: Improved n=15, Non-improved n=31; available-case analysis)*

| **Variable** | **Improved (ΔEF ≥10%; n=15)** | **Non-improved (ΔEF <10%; n=31)** | **p-value** | **N (Imp/Non)** |
| --- | --- | --- | --- | --- |
| Baseline LVEF, % | 26.6 [18.8–32.3] | 29.0 [25.5–35.6] | 0.106 | 15/31 |
| E/e′ | 15.7 [10.5–22.2] | 11.1 [9.7–13.3] | 0.048 | 14/28 |
| LV s′ | 4.8 [3.7–5.2] | 5.5 [4.7–6.7] | 0.101 | 14/28 |
| LV mass index | 108.0 [93.3–130.1] | 120.8 [109.5–143.0] | 0.087 | 15/31 |
| Relative wall thickness | 0.4 [0.3–0.5] | 0.4 [0.3–0.4] | 0.313 | 15/31 |
| Left atrial volume index | 46.9 [36.3–49.2] | 34.9 [29.8–44.0] | 0.079 | 15/31 |
| Deceleration time | 184.8 [150.6–194.0] | 192.3 [138.5–221.8] | 0.566 | 14/28 |
| LVEDD | 48.2 [45.3–55.5] | 53.7 [49.5–57.5] | 0.071 | 15/31 |
| LVESD | 41.7 [38.4–48.0] | 45.0 [42.5–49.5] | 0.261 | 15/31 |
| Preop creatinine | 1.0 [0.8–1.2] | 1.1 [0.9–1.6] | 0.981 | 15/31 |
| Preop GFR | 61.0 [53.0–77.0] | 71.0 [44.5–92.5] | 0.582 | 15/31 |
| Male | 10/15 (66.7%) | 26/31 (83.9%) | 0.257 | 15/31 |
| Diabetes | 8/15 (53.3%) | 20/31 (64.5%) | 0.530 | 15/31 |
| Hyperlipidemia | 7/15 (46.7%) | 8/31 (25.8%) | 0.191 | 15/31 |
| Smoking (current/ever) | 2/15 (13.3%) | 5/31 (16.1%) | 1.000 | 15/31 |
| CVA/TIA | 2/15 (13.3%) | 5/31 (16.1%) | 1.000 | 15/31 |
| Atrial fibrillation | 4/15 (26.7%) | 2/31 (6.5%) | 0.078 | 15/31 |
| Prior PCI | 3/15 (20.0%) | 12/31 (38.7%) | 0.317 | 15/31 |
| # diseased vessels | 3.0 [3.0–3.0] | 3.0 [3.0–3.0] | 0.611 | 15/31 |
| Age | 69.0 [65.0–78.5] | 67.0 [62.0–76.5] | 0.467 | 15/31 |

Values are median [IQR] or n/N (%). p-values were calculated using the Mann–Whitney U test for continuous variables and Fisher’s exact test for categorical variables. Available-case analysis was used; denominators vary by variable. The subgroup was defined from the corrected outcome-analysis cohort after excluding patients without an evaluable early postoperative LVEF.
